# A Case of Thanatophoric Dysplasia Type I with Fetal Hydrops in the First Trimester

**DOI:** 10.1155/2016/1821230

**Published:** 2016-02-23

**Authors:** Giannina Calongos, Masateru Hori, Mai Ogino, Hideaki Sawai

**Affiliations:** ^1^Department of Obstetrics and Gynecology, Meiwa General Hospital, Nishinomiya 663-8186, Japan; ^2^Department of Obstetrics and Gynecology, Hyogo College of Medicine, Nishinomiya 663-8501, Japan

## Abstract

During a routine prenatal exam, a 36-year-old female in her third pregnancy was diagnosed with fetal hydrops at 11 weeks of gestation. The pregnancy was monitored with periodic ultrasounds; however, spontaneous resolution was not observed. Amniotic fluid examination at 16 weeks of gestation showed a normal karyotype; however, macrocephaly, a narrow thorax, and shortening of the long bones were observed on ultrasonography. With the strong suspicion of a fetal skeletal disease, specifically thanatophoric dysplasia (TD), and after extensive genetic counseling, termination of the pregnancy was performed per the parents' wishes with mechanical cervical dilation and gemeprost (PGE1) administration. Following delivery, the fetus was found to have macrocephaly, a narrow bell-shaped thorax, and a protuberant abdomen, as well as curved long bones, H-shaped platyspondyly, and curved clavicles on skeletal radiography. As a result, the fetus was diagnosed with TD type I. This case illustrates that although TD is a rare disease, an accurate prenatal diagnosis can be made with the use of ultrasonography.

## 1. Introduction

Thanatophoric dysplasia (TD) is a rare and lethal skeletal dysplasia with an estimated incidence of 1 in 20000 to 40000 births [1] and was first described by Maroteaux et al. in 1967 [2]. TD can be classified into two types: type I is characterized by micromelia with bowed femurs and, uncommonly, the presence of cloverleaf skull deformity; type II is characterized by micromelia with straight femurs and moderate to severe cloverleaf skull deformity [3]. A common feature to both types is the presence of a narrow thorax which causes respiratory failure shortly after birth [4, 5].

Fetal hydrops is defined as an abnormal fluid collection in two or more areas of the fetal body. It can be further classified as immune or nonimmune. Immune fetal hydrops develops due to fetal hemolysis secondary to incompatibility of maternal and fetal blood type. Nonimmune hydrops, on the other hand, can result from a large number of causes [6, 7]. However, 20–30% of hydrops is of unknown etiology.

Here, we report a case of TD type I with fetal hydrops diagnosed in the first trimester and thereafter shortening of the long bones and macrocephaly. Confirmation of the diagnosis was made by clinical examination and radiologic studies after delivery.

## 2. Case Presentation

**Figure 1:**
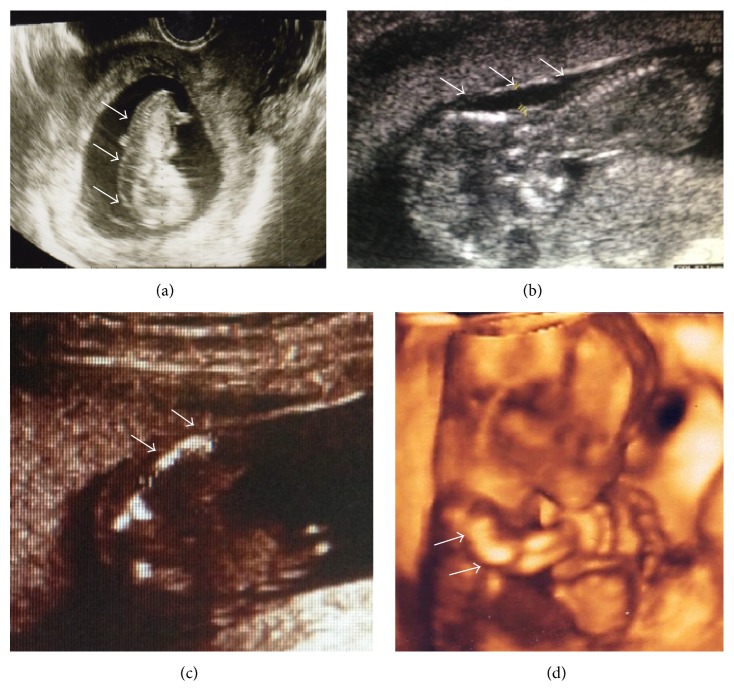
Fetal ultrasound with skin edema in the posterior part of the head and vertebral column (→) (a). A longitudinal view of the head and the thorax demonstrating skin edema in the posterior part of the neck (→), macrocephaly, and narrow thorax (b). Fetal ultrasound showing short curved femur (→) (c). A three-dimensional image with a view of the upper limbs showing bowed humerus (→) (d).

A 36-year-old female in her third pregnancy came to our hospital at 8 weeks of gestation for a routine prenatal check. The couple and their previous children's past and family histories were unremarkable. Ultrasonographic examination at 8 weeks of gestation was unremarkable, with a crown-rump length (CRL) of 15.9 mm. At 11 weeks of gestation, although the CRL was 49.6 mm corresponding to gestational age, fetal hydrops was evident on ultrasonography (Figure 1(a)). Since screening for immune hydrops and congenital infections were negative, a transvaginal ultrasound was performed the following week for further evaluation. At 12 weeks of gestation, appropriate fetal growth was observed with a CRL of 60 mm; however, fetal hydrops was still present. The couple was counseled on the different etiologies of fetal hydrops; however, at this point the cause was not clear.

**Figure 2:**
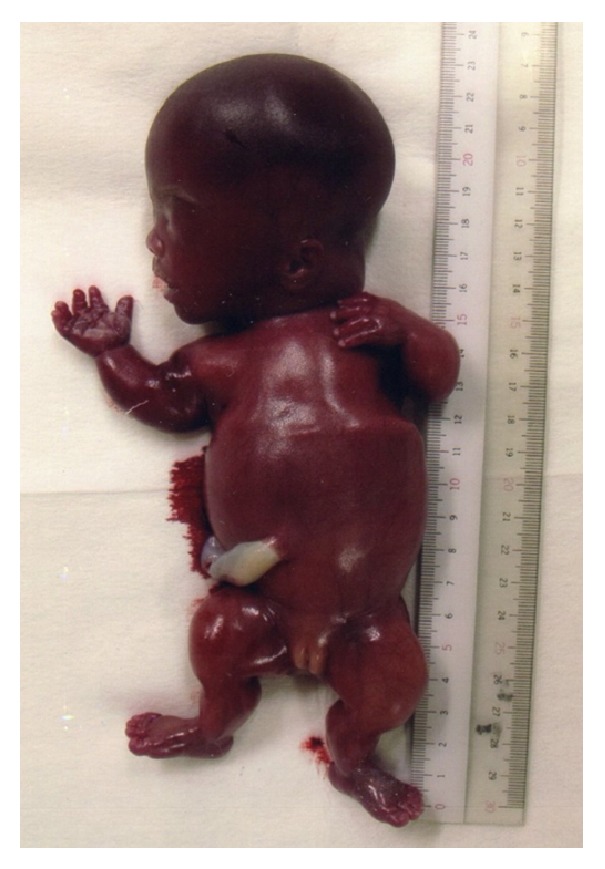
Female fetus of 20 weeks of pregnancy with short limbs and redundant skin folds. Also, macrocephaly, narrow bell-shaped thorax, and protuberant abdomen were noticed. Skull and facial characteristics were normal.

**Figure 3:**
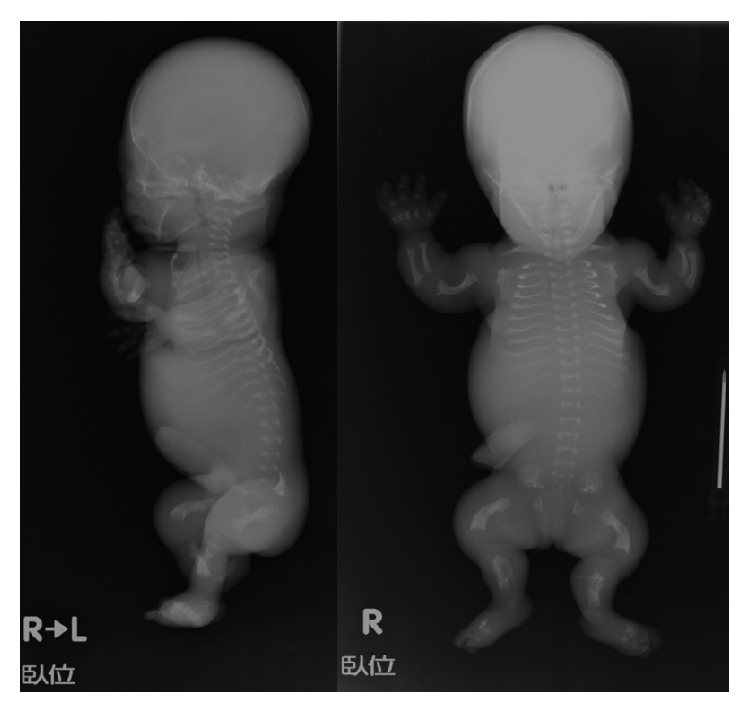
Anteroposterior and lateral radiographs of the fetus showing telephone receiver-like curved femurs and humeri with irregular metaphyses, H-shaped platyspondyly, and curved clavicles. No cloverleaf skull deformity was observed.

At 13 weeks of gestation the biparietal diameter (BPD) was measured to be 27.4 mm, corresponding to the 95th percentile. From this week on macrocephaly was observed and a narrow thorax was suspected on subsequent ultrasonographies (Figure 1(b)). At 16 weeks of gestation, amniotic fluid examination showed a normal karyotype (46XX); however, a routine and a four-dimensional ultrasound revealed shortening and bowing of the long bones (femur length (FL) 11.6 mm) (Figures 1(c) and 1(d)) with no subsequent improvement. As a result, a fetal skeletal disease, specifically TD, was strongly suspected. At 20 weeks of gestation, the FL was 1.18 cm, compatible with less than the 5th percentile. Also, the BPD was 5.3 cm, corresponding to more than the 95th percentile. During genetic counseling, a molecular analysis was suggested to the parents in order to make an accurate prenatal diagnosis of TD; however, they opted not to pursue this exam and decided on termination of the pregnancy, instead. The patient was then hospitalized and underwent mechanical cervical dilation. The following day gemeprost (PGE1) was administered intravaginally every three hours. A 400 g female fetus was delivered dead at 20 weeks and 2 days of gestation (Figure 2). All limbs were noted to be extremely short with redundant skin folds. Macrocephaly was evident. A narrow bell-shaped thorax with short ribs and a protuberant abdomen were noticed; however, the skull and facial characteristics were within normal limits. Skeletal radiography showed telephone receiver-like curved femurs and humeri accompanied by irregular metaphyses, an H-shaped platyspondyly, and curved clavicles (Figure 3). No cloverleaf skull deformity was observed. These characteristics confirmed the diagnosis of TD type I.

## 3. Discussion

The incidence of immune hydrops has decreased due to routine screening and prophylaxis; however, the mortality rate of nonimmune hydrops, during either the fetal or the neonatal period, is up to 75.5% [8]. Although fetal hydrops is considered to be a nonspecific finding on obstetric ultrasounds, previous reports showed that an increased nuchal translucency (NT) and hydrops are common features of serious skeletal dysplasia [9]. Moreover, previous cases of TD reported a NT of 3.4–6.5 mm by 14 weeks of gestation which correlates with the 5.7 mm observed in this case [9].

As previous studies reported, 40–80% of TD cases can be correctly diagnosed by ultrasonography in the prenatal period [10]. Limb shortening in TD is also sonographically apparent from as early as 13 weeks of gestation. Similarly, head circumference (HC) is increased throughout gestation, a feature that is present as early as the first trimester [10]. With the evidence of short limbs, a hypoplastic thorax, and macrocephaly, we strongly suspected a skeletal disease as TD.

Published reports have used a femur length/abdominal circumference (FL/AC) ratio <0.16 as a predictor for lethal skeletal dysplasia [11]. Also, a hypoplastic thorax is suspected with a thoracic circumference of less than 5% at the level of the four-chamber view of the heart or a thoracic-AC ratio of less than 0.79 [12]. In this case the FL/AC ratio was 0.103 (0.82 cm/7.9 cm) and 0.0769 (1.18 cm/15.34 cm) at 13 and 20 weeks of pregnancy, respectively. A narrow bell-shaped thorax was suspected as early as 13 weeks of pregnancy.

TD is the most frequent lethal skeletal dysplasia caused by mutation of the fibroblast growth factor 3 (FGFR3) gene [13, 14]. Although molecular analysis of fetal cells to detect Arg248Cys, Tyr373Cys, Lys650Glu, and other specific mutations enables an accurate prenatal diagnosis of TD [1], it was not performed in this case per the couple's wishes. However, since limb shortening is associated with a 2.7 relative risk for trisomy 21 [4] and a case of TD type I presented with trisomy 21 was reported previously [15], we considered an amniocentesis at 16 weeks of gestation important to perform. TD is an autosomal dominant genetic disease; however, it is almost always caused by a de novo mutation in FGFR3 [3]. As a result, a general empiric recurrence risk is estimated in only 2% [16]. This fact is important to consider in order to relieve parental anxiety over future pregnancies. After counseling, the patient had a fourth pregnancy without complications and delivered a normal female baby at term.

In summary, even though TD is a rare condition, ultrasonography can be used to obtain an accurate prenatal diagnosis and facilitate early parental counseling.
